# Lineage isolation in the face of active gene flow in the coastal plant wild radish is reinforced by differentiated vernalisation responses

**DOI:** 10.1186/s12862-016-0655-7

**Published:** 2016-04-16

**Authors:** Qingxiang Han, Hiroyuki Higashi, Yuki Mitsui, Hiroaki Setoguchi

**Affiliations:** Graduate School of Human and Environmental Studies, Kyoto University, Yoshida Nihonmatsu-cho, Sakyo-ku, Kyoto Japan; Faculty of Agriculture, Tokyo University of Agriculture, Funako 1737, Atsugi, Kanagawa Japan

**Keywords:** Demographic history, Gene flow, Isolation-with-migration model, Kuroshio Current, Lineage differentiation, Natural selection, Vernalisation, Wild radish

## Abstract

**Background:**

The respective role and relative importance of natural selection and gene flow in the process of population divergence has been a central theme in the speciation literature. A previous study presented conclusive evidence that wild radish on Japanese islands comprises two genetically isolated lineages: the southern and northern groups. However, a general understanding of the lineage isolation with frequent seed flow of the coastal plant species is still unclear. We surveyed nucleotide polymorphisms over 14 nuclear loci in 72 individuals across the Japan–Ryukyu Islands Arc to address the demographic history of wild radish utilising the isolation-with-migration (IM) model. In addition, we investigated the flowering times of individuals in different wild radish lineages, with and without cold exposure, to assess their respective vernalisation responses.

**Results:**

Coalescent simulations suggested that divergence between the southern and northern lineages of wild radish began ~18,000 years ago, initially during the Last Glacial Maximum (LGM) period. The gene flow from the southern to northern groups was considerably higher than that in the opposite direction, indicating effective dispersal of viable seeds via the northward Kuroshio Current. Our greenhouse experiments indicated that cold exposure was not required for flowering in the southern group, but could advance the date of flowering, suggesting that vernalisation would be facultative in the southern group. In contrast, the northern group was either unable to flower or flowered later without prior cold exposure, and thus had an obligate requirement for cold treatment.

**Conclusions:**

The south–north lineage divergence in wild radish could be triggered by a directional change in the sea current during the ice age, despite gene flow due to the high dispersability and longevity of seeds. We also found that temperature profoundly affected the vernalisation responses of wild radish, which may repress reproductive success and ultimately drive and reinforce intra-specific differentiation between the two lineages of wild radish. This study provides new insights into the maintenance of lineage differentiation with on-going gene flow in coastal plants.

**Electronic supplementary material:**

The online version of this article (doi:10.1186/s12862-016-0655-7) contains supplementary material, which is available to authorized users.

## Background

Divergence (and ultimately, speciation) refers to the splitting of a single ancestor into two or more descendant lineages. Divergence of a population is a tug-of-war between the forces that generate differences, such as natural selection, and the main force that erodes differences, gene flow [1]. Therefore, to fully understand speciation, one must understand the respective roles and relative importance of natural selection and gene flow in the process of divergence that leads to speciation. Divergent natural selection could shape a barrier that reduces gene flow owing to adaptations of populations to divergent habitats. As sessile organisms, plants are obliged to adapt to their habitat in order to thrive. In these habitats, flowering time is a critical trait for adaptation to diverse natural environments, because it synchronises reproduction for seed production in the most favourable season of the year. In addition, it determines both the time invested for vegetative growth and the resources available for the reproductive phase, which are particularly important in agriculture in terms of productivity. Flowering time is strongly affected by the ambient temperature, particularly low temperatures; this is known as vernalisation [2]. Specifically, vernalisation is defined as the exposure of a plant to low temperatures that induce or promote the subsequent development of floral primordia [3]. The vernalisation responses of plants can be categorised as obligate or facultative [4]. Plants with obligate vernalisation require cold treatment to acquire the ability to flower; in those with facultative vernalisation, cold treatment accelerates flowering or improves flowering characteristics. Different vernalisation responses with varying subsequent flowering times may lead to a reduced rate of mating between native and immigrant individuals, thereby facilitating divergence between populations in different habitats.

The magnitude of gene flow plays a critical role in the context of speciation, because it determines the extent to which populations diverge from one another. The absence of gene flow contributes to population differentiation. In contrast, high levels of gene flow may homogenise genes responsible for divergence and thus act as a hindering force on divergence. This is particularly true in coastal plants such as *Calystegia soldanella* [5, 6], *Lathyrus japonicas* [7], *Uniola paniculata* [8], and *Carex arenaria* [9]. Species-specific biological properties of coastal plants have been proposed to be the major cause of this phenomenon. *Cakile maritima* seeds reportedly have 92 buoyancy for 1 year and 80 % seed germination for 4 months [10], and *C. soldanella* are thought to retain 90 % buoyancy for up to 27 months and 90 % seed germination capacity after immersion in seawater for 12 months [5]. These findings indicate that seeds of coastal plants exhibit high dispersability and longevity, which explain their long-distance dispersal, and that substantial gene flow slows population divergence in coastal plants. Accordingly, divergence is considered to be difficult, particularly in the presence of gene flow between lineages [11].

The genus *Raphanus* (Brassicaceae) is a globally important root vegetable crop. Apart from the cultivated radish, there are several wild species of *Raphanus*. Among these, *R. raphanistrum*, including subsp. *maritium*, subsp. *raphanistrum* and subsp. *landra*, is distributed throughout Eurasia, Africa and North America [12]. However, another wild radish species, *R. sativus* var. *raphanistroides* Makino, is widely distributed in sandy coastal areas of East Asia [13]. The East Asian wild radish (hereafter, wild radish) is a winter annual, self-incompatible and insect-pollinated coastal plant. The life history and morphological traits of the East Asian wild radish are similar to those of cultivars. However, unlike most cultivars, it does not develop an edible succulent root. In fact, it was once thought that the Japanese wild radish was an escapee of cultivated radishes [14]. However, this proved incorrect following studies of mitochondrial DNA [15], and the origin of the East Asian wild radish remains in question. A recent comparative study of chloroplast (cp) DNA variability showed that Japanese wild radish accessions share common haplotypes with cultivars, suggesting incomplete lineage sorting and/or multiple origins of the cultivars [13]. This shows the close relationship between wild radish and cultivars, and suggests that increased understanding of the characteristics of wild radish would contribute greatly to breeding and evolutionary studies of radish cultivars.

Wild radish has several advantages for basic research in genetics and molecular biology. Its genome has been largely sequenced and annotated [16], its life cycle is short (ca. 2–3 months from germination to anthesis), it produces seeds prolifically, is easily cultivated in restricted spaces, and is closely related to *Arabidopsis*, to which it likely has considerable genetic homology. In coastal plants, evidence of divergence between lineages remains sparse due to the high dispersability and longevity of seeds; however, a previous study using nine microsatellites presented conclusive evidence that wild radish on Japanese islands comprises two genetically isolated lineages, namely, the southern and northern groups [17]. However, a general understanding of the relationships of divergence and the counteracting factors of coastal plant populations is still unclear. To address this question, advanced methods of evolutionary modelling are beneficial for determining the demographic history of coastal plants. In particular, the isolation-with-migration (IM) model can evaluate the presence or absence of gene flow and its direction and strength [18, 19]. The application of this model may help clarify the relationships between genetic divergence and gene flow in coastal plants. To date, this common but reliable and effective approach has not been applied to wild radish.

Here, we utilised the IM model for estimating the demographic history of wild radish in southern and northern regions of Japan, regarding the divergence time of the two lineages, the rates and directions of gene flow between lineages, and their respective effective population sizes. In addition, we assessed the vernalisation responses to local temperature in different wild radish groups, and attempted to clarify the influence of local adaptation on population divergence. Finally, we clarified the effect of interplay between natural selection and gene flow on divergence. This study will provide new insights into the process of lineage differentiation in spite of gene flow in coastal plants, particularly those that are unique to wild radish.

## Methods

### Sampling and DNA extraction

**Fig. 1:**
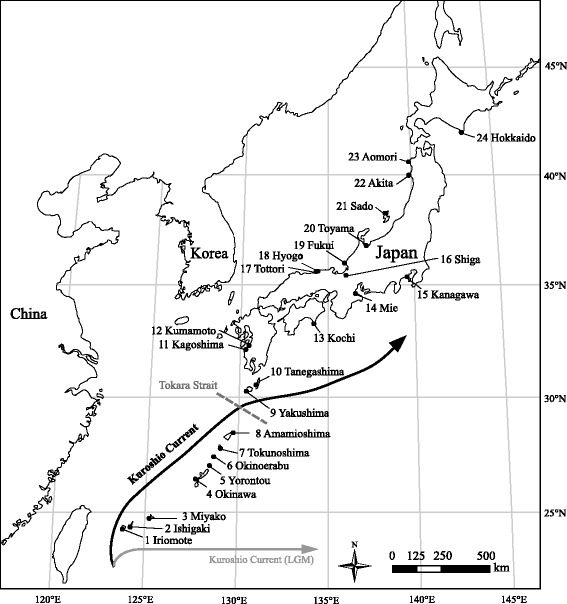
Map showing the sampling locations. The main routes of the Kuroshio Current in the present (black line) and during the LGM (grey line) are adapted from the graph of Ujiié & Ujiié [47]. The dotted line shows the location of Tokara Strait. Population codes correspond to those in all tables and figures

Leaf materials of wild radish were collected from 24 natural populations, covering almost the entire geographic range of Japan (Fig. 1). We randomly selected 3 individuals per site, and a total of 72 individuals were used. As wild radish is a common and widely distributed coastal plant, neither ethical approval nor specific permission to conduct sampling was required in this study. Genomic DNA was extracted and purified from silica gel-dried leaves using the cetyltrimethylammonium bromide (CTAB) method [20], and stored at 4 °C until further processing.

### Polymerase chain reaction (PCR) amplification and sequencing

Primers were designed for the flanking exonic regions based on the genomic sequences of cultivars [16] using Primer 3 software [21] (http://primer3.ut.ee/). Initial sequencing was conducted for 34 nuclear loci, including *COL*, *PHY*, *VEL*, *MYB*, *MYB*, *FT*, *FLC*, *FRI*, *GL1*, *GL3*, *CRY*, *LFY*, *TBL*, *CHI*, *CHS*, *FPF* and *RTV*. To avoid background noise in the sequence data, 17 new internal primers were designed that were more specific for wild radish samples. Finally, a set of fourteen unequivocal and reliable nuclear loci were selected and sequenced (Additional file 1: Table S1).

The PCR mixture (10 μL) contained 50 ng genomic DNA, 0.2 mM dNTP mixture, 0.25 U Taq polymerase (Takara), 10× Ex Taq Buffer (Takara Ex Taq; Takara, Kusatsu, Japan), 0.2 μM each primer, and sterile water. The reaction proceeded via initial denaturation at 94 °C for 5 min, 35 cycles of 94 °C denaturation for 1 min, 55 °C annealing for 1 min, 72 °C extension for 1.5 min, and a final 72 °C extension for 10 min. After purification using ExoStar (GE Healthcare Ltd., Little Chalfont, Buckinghamshire, UK), PCR products were directly sequenced from both directions using the BigDye Terminator Cycle sequence Ready Reaction Kit ver. 3.1 (Applied Biosystems, Foster City, CA, USA). Alignments were performed on an Auto Assembler (Applied Biosystems).

### Nucleotide diversity and neutrality tests

The haplotypes of all of the samples were probabilistically determined using PHASE with default settings in DnaSP ver. 5.10 [22]. The number of segregation sites (*S*), the average number of pairwise nucleotide differences per site (*π*) [23], the haplotype diversity (*H*_d_) and the extent of nucleotide polymorphisms in terms of *θ*_w_ [24] were calculated for each locus. The minimum number of recombination events (*R*_*m*_) within the 14 loci was estimated using the four-gamete test [25] with DnaSP ver. 5.10. Generally, Tajima’s *D* [26] and Fu and Li’s D* [27] are more effective than the commonly used single test for detecting balancing selection, whereas the multi-locus Hudson-Kreitman-Aguade (HKA) test [28] is more powerful than Tajima’s *D* and Fu and Li’s D* tests for detecting selective sweeps [29]. Hence, this study applied a combination of these tests to provide the most likely mechanism causing any departure from neutrality. Tajima’s *D* and Fu and Li’s D* and F* were estimated using DnaSP ver. 5.10. In addition, the HKA test was performed using the HKA program (http://lifesci.rutgers.edu/heylab/HeylabSoftware.htm). The significance of each test was determined using 1000 coalescence simulations.

### Population structure

A model-based Bayesian clustering method was used to estimate the geographic structuring within species using the software STRUCTURE 2.3 [30]. The model parameters were set to “admixture” with independent allele frequencies among populations, and 25 replicate runs were performed for each value of *K* with 5 × 10^5^ iterations after 10^5^ iterations as a burn-in period. After removing questionable runs as outliers, 20 of 25 runs were ultimately used for subsequent analysis. The optimal number of clusters was determined using the *∆K* criterion [31]. Principal coordinate analysis (PCoA) was conducted based on Nei’s genetic distance [32] matrices between populations, using GenAlEx 6.5 [33] and R-package [34].

### IM analysis

To estimate the demographic parameters, regarding the divergence time (*T*) of different lineages (based on the results of Structure analysis), bidirectional migration rate (*m*_S>N_ and *m*_N>S_), and the effective population size of the ancestral (*θ*_A_) and descendant population (*θ*_S_ and *θ*_N_), we employed the IM model [18, 19] and Markov chain Monte Carlo (MCMC) method using the IMa2 program [35, 36]. After several preliminary runs to optimise the prior boundaries for the primary six demographic parameters (*T*, *m*_S>N_, *m*_N>S_, *θ*_A_, *θ*_S_ and *θ*_N_), each IM simulation was conducted for 10^6^ MCMC steps with a burn-in of 10^5^ steps, and Metropolis-coupling of 120 independent heated chains. To select proper models of nucleotide substitution for IMa2 analyses, the infinite-sites (IS) mutation model was evaluated for each locus [37]. The mixing properties of MCMC were assessed by monitoring the effective sample size (ESS), the swapping rates between successive chains of MCMC, and the trend-line plots of the parameters. When independent runs produced similar posterior distributions, well-mixed runs were repeated to get reproducible results. Three independent runs were performed with different numbers of seeds to guarantee convergence of samples [19, 38]. The lowest ESS among the parameters was above 50, to ensure adequate mixing and convergence. These analyses were performed using the largest non-recombination block for each locus.

After all of the runs were converged under the full model, which estimated the six demographic parameters, the fit of data to simple demographic models was tested using the nested model approach in the Load-Genealogies model [35]. A total of 3 × 10^5^ genealogies were used to calculate log-likelihood ratio statistics for possible nested models, the significance of which was assessed using a Chi-squared (*χ*2) test. We compared log-likelihoods of full and nested models in 24 different divergence models by setting different gene flow and population size parameters. To correct for multiple hypothesis testing, the Benjamini-Hochberg method [39] was applied with a false discovery rate of 5 % using the R function ‘*p*.adjust’. Demographic parameters were scaled using the geometric mean of the 7 × 10^−9^ substitutions/site/year for nuclear loci in *Arabidopsis thaliana* [40, 41].

### Flowering time and temperature

Seeds of wild radish in southern (1 Iriomote and 4 Okinawa Islands) and northern regions (16 Shiga, 14 Mie, 23 Aomori prefectures and 24 Hokkaido Islands) were collected (see Fig. 1 for population locations). Eight individuals per site were simultaneously cultivated in two conditions, with or without 3 weeks of cold treatment at 5 °C. Seeds were sown in Petri dishes containing water-soaked filter paper in the dark at 21 °C for 3 days. Then the seedlings were transplanted to 42 mm diameter Jiffy pots (Nippon Jiffy Pot Products, Yokohama, Japan), and placed within the growth chamber (21 °C, 10 h of light/14 h of darkness) for vegetative development for 1 week. For the vernalisation group, 1-week-old seedlings experienced cold treatment at 5 °C (10 h of light/14 h of darkness). After 3 weeks of vernalisation treatment, plants were transplanted into plastic pots (diameter 9 cm × height 20 cm). Subsequently, the pots were moved to an air-conditioned greenhouse (21 °C) in Kyoto (35°01′N/135°46′E). For the non-vernalisation group, samples were planted in pots and subsequently moved to the same greenhouse for 1 week after the germinated seedlings were fully developed. Flowering time was measured as days to flowering (i.e., the number of days from planting in the greenhouse until the appearance of the first flower). The experiment was finished 120 days after the wild radishes were planted in the greenhouse.

A statistical analysis was performed using a one-way analysis of variance (ANOVA) followed by the Student’s two-tailed *t*-test for paired comparisons with SPSS ver. 23.0 software (SPSS Inc., Chicago, IL); *p*-values less than or equal to 0.05 were considered to indicate statistical significance. To investigate the possible relationships between temperature and vernalisation responses in wild radish, the variability of temperature during the coldest month in Japan was obtained through publicly available datasets. Temperature values were extracted to grid points (the highest resolution: 2.5 min from WorldClim, www.worldclim.org). Current conditions involved interpolations of observed data representative of the years 1950–2000, whereas past conditions were representative of 22,000 years ago. A map of the minimum temperature of the coldest month (current and past conditions) in Japan was produced using ArcGIS version 10.3 (ESRI, Redlands, CA, USA).

## Results

### Nucleotide variation and neutrality tests

The sequenced fragments ranged from 235 base pairs (bp) to 577 bp with a total concatenated length of 5756 bp, excluding gaps and missing data (Table 1). Insertion-deletion (indel) polymorphism was found in each of *TBL19* and *CRY2*, but these were excluded from subsequent analyses. The nucleotide diversity at each locus is presented in Table 1. The levels of nucleotide polymorphism (*θ*_w_ and *π*) differed between loci; *LFY* was the most polymorphic locus (*θ*_w_ = 0.00847), and *TBL19* was the least polymorphic (*θ*_w_ = 0.00179). Estimates of total nucleotide variation ranged from 0.00179 to 0.00847 for *θ*_w_, with an average of 0.00417. Compared to other plants in the Brassicaceae, the nucleotide diversity of wild radish (self-incompatibility) is close to that of *Pugionium cornutum* (self-incompatibility; mean *θ*_w_ = 0.00502) and *Pugionium dolabratum* (self-incompatibility; mean *θ*_w_ = 0.00576), as revealed by eight nuclear loci [42], and notably higher than that of *Thellungiella salsuginea* (self-compatibility), as observed from ten nuclear loci (mean *θ*_w_ = 0.00036) [43]. The mean nucleotide diversity per site (π) within populations was 0.00612 (0.00209 to 0.01271). The haplotype diversity (*H*_d_) ranged from 0.372 (*COL4*) to 0.708 (*VEL2*), with an average of 0.554; this was relatively low compared to *P. cornutum* (mean *H*_d_ = 0.853) and *P. dolabratum* (mean *H*_d_ = 0.903) [42]. The minimum number of recombination events (*R*_*m*_ ≤ 5) was detected by the four-gamete test at most loci, whereas intra-locus recombination in *PHYA*, *PHYC*, and *COL5* was high. To exclude the influence of recombination, the maximum non-recombined regions were extracted from all of the 14 loci and subjected to IMa2 analyses.

**Table 1:** Summary of the nucleotide polymorphisms and neutrality tests of fourteen loci for wild radish

Locus	Aligned size (bp)	No. of seqs.	Largest non-recombining blocks (bp)	*R*_*m*_	*S*	*θ*_w_	*π*	*N*_h_	*H*_d_	Neutrality tests		
										*D*	D*	F*
*PHYA*	473	140	149	8	5	0.00608	0.01008	4	0.610	1.32690	1.00882	1.31890
*PHYB*	420	142	151	6	5	0.00599	0.01271	6	0.506	2.26059*	−0.09887	0.81392
*PHYC*	480	134	236	2	6	0.00465	0.00669	7	0.635	0.94466	1.09653	1.23822
*PHYE*	378	144	260	2	4	0.00278	0.00654	4	0.583	2.53131*	0.91024	1.70217
*TBL19*	415	134	347	2	3	0.00179	0.00339	4	0.537	1.52497	0.80285	1.21693
*TBL21*	559	140	175	5	3	0.00311	0.00576	4	0.517	1.44525	−0.65975	0.01794
*MYB29*	441	140	441	0	5	0.00206	0.00527	4	0.658	3.16093**	1.00882	2.04191**
*COL4*	577	136	280	4	4	0.00325	0.00374	5	0.372	0.30524	−1.19153	−0.82014
*COL5*	357	140	135	6	4	0.00537	0.00361	4	0.459	−0.61567	−2.83513*	−2.48960*
*CHI*	366	144	274	2	3	0.00198	0.00209	4	0.536	0.09504	−0.66570	−0.49647
*VEL2*	317	142	196	2	3	0.00277	0.00498	4	0.708	1.34849	0.79831	1.14817
*LFY*	235	142	235	0	11	0.00847	0.00281	5	0.494	−1.66219	−3.50335**	−3.38634**
*CRY2*	406	132	242	5	9	0.00682	0.01042	5	0.696	1.26908	1.30095	1.53412
*CRY3*	332	132	112	3	2	0.00327	0.00760	3	0.441	1.93184	0.66462	1.24454
Mean	411	139	231	3.4	4.8	0.00417	0.00612	4.5	0.554	-	-	-

*R*_*m*_ estimate of minimum number of recombination events, *S* number of polymorphic sites, *θ*_w_ Watterson’s estimator of *θ* per base pair*, π* nucleotide diversity, *N*_h_ number of haplotypes, *H*_d_ haplotype diversity, *D* Tajima’*D* and Fu & Li’s D* and F*

Significant levels: *, 0.01 ≤ *p* < 0.05; **, *p* < 0.01

The three neutrality tests yielded contrasting results. With regard to the single-locus neutrality tests, only three loci (*PHYB*, *PHYE*, and *MYB29*) yielded significant values (*p* < 0.05) for Tajima’s *D*, whereas Fu and Li’s D* was significant for two loci (*COL5* and *LFY*) and Fu and Li’s F* showed significant values for two other loci (*MYB29* and *LFY*). Overall, none of the 14 loci showed a consistently significant deviation from neutral expectation for Tajima’s *D* and Fu and Li’s D* and F* statistics. For the HKA test, none of the loci significantly departed from the neutral equilibrium model and thus all of the 14 loci were used for the subsequent analyses.

### Population genetic structure

**Fig. 2:**
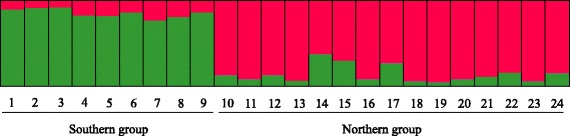
Individual assignment per population, as given by structure analysis for *K* = 2. The population numbers are shown beneath the bars

**Fig. 3:**
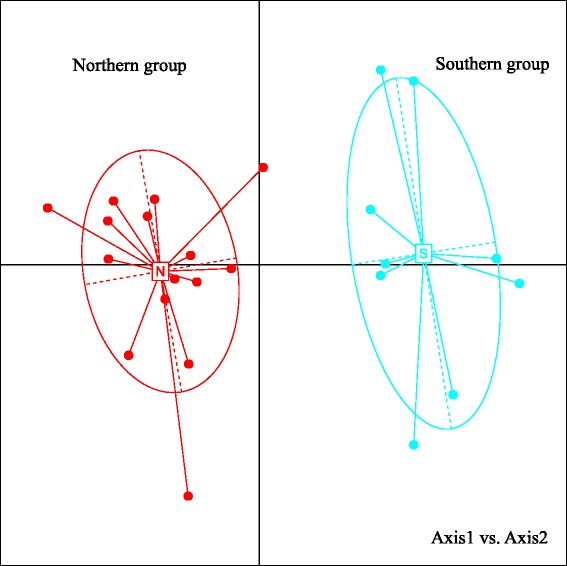
Principal coordinate analysis of 24 populations of wild radish in Japan based on their genetic distances. N: northern lineage; S: southern lineage

The most likely number of clusters across all individuals was *K* = 2 (Additional file 2: Figure S1), measured by the ∆*K* statistic. The structure analysis revealed significant differentiation in sibling populations of wild radish: the southern (pops. 1–9) and northern (pops. 10–24) lineages (Fig. 2) agreed strongly with previous findings [17]. These populations entirely corresponded to separate geographic regions. Groups on the Yakushima Islands (pop. 9) and other southern populations were clustered into the southern lineage, whereas the neighbouring Tanegashima (pop. 10) and northern populations constituted the northern lineage. Signs of genetic admixture between the southern and northern lineages were observed in a few individuals. The population-based PCoA agreed with the output of structure, also revealing two major genetic groups, the southern and northern lineages (Fig. 3). The first and second axes extracted 32.01 and 15.74 % of the total genetic variation, respectively.

### Demographic history

**Fig. 4:**
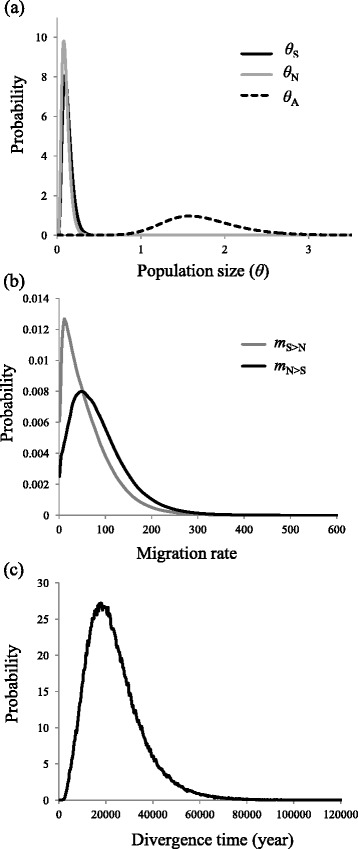
Marginal distribution of the posterior probabilities of demographic parameters estimated by IMa2 analysis : (**a**) *θ*_A_, *θ*_S_and *θ*_N_ are the effective population sizes of ancestral, southern and northern populations, respectively; (**b**) *m*_S>N_ and *m*_N>S_ are the gene flow from northern to southern lineage and southern to northern lineage, respectively; (**c**) the divergence time between southern and northern linage

We performed multiple IM simulation runs for the two wild radish lineages obtained in structure analysis, using IMa2 to detect the marginal posterior distributions of the probabilities of the six demographic parameters (*T*, *m*_S>N_, *m*_N>S_, *θ*_A_, *θ*_S_, and *θ*_N_). Independent runs in the M-model gave consistent maximum likelihood estimates (MLEs), indicating the robustness of these values for the demographic parameters. MLEs and the 95 % highest probability density (HPD) of demographic parameters of the IM model are summarised in Table 2, and the marginal distributions of the probabilities of the parameters are shown in Fig. 4, with a single peak posterior probability for each parameter. The estimated effective population size was 0.094 (95 % HPD = 0.030–0.246) in the southern region and 0.082 in the northern region (95 % HPD = 0.026–0.200), as shown in Table 2. The peak of the posterior distribution of the ancestral effective population size for southern and northern groups was less pronounced across a broad range of priors. Analyses of the full dataset indicated nonzero and significant bidirectional gene flow between the populations. The IM simulations rejected the strict isolation model of population divergence and provided evidence of bidirectional but asymmetrical gene flow between the two groups. The estimated migration rate from south to north (MLE: 51) was effectively higher than that in the opposite direction (MLE: 11), corresponding to population migration rate estimates (2 Nm) of 3.188 and 1.351 migrants per generation, respectively (Table 2). The sizes of the two daughter populations were considerably smaller than the ancestral population (*θ*_N_ = 1.582, 95 % HPD = 0.914–2.642; Table 2), indicating that wild radish underwent strong population reductions after divergence. The scaled divergence time for the southern and northern populations was ca. 18,000 years ago (95 % HPD = 4173–47237) during the Last Glacial Maximum (LGM; ca. 18,000–22,000 years ago). Table 3 shows the likelihood ratio statistics for a series of nested speciation models applied to the full dataset. Adjustment for multiple testing via the Benjamini-Hochberg process yielded significant *p*-values at a false discovery rate of 5 % for all 24 models. Thus, all of the nested models in which the two migration rates were zero or equal to each other (*m*_S_ = 0, *m*_N_ = 0, *m*_S_ = *m*_N_ = 0, *m*_S_ = *m*_N_), and those in which the two effective population sizes were equal to each other or the ancestral population (*θ*_S_ = *θ*_N_, *θ*_A_ = *θ*_S_, *θ*_A_ = *θ*_N_, *θ*_A_ = *θ*_S_ = *θ*_N_) were rejected.

**Table 2:** Maximum-likelihood estimates (MLEs) and the 95 % highest posterior density (HPD) intervals of demographic parameters from pairwise IMa2 multi-locus analyses

	*θ*_S_	*θ*_N_	*θ*_A_	*m*_N>S_	*m*_S>N_	2N_N_m_N_	2N_S_m_S_	*t*	*T* (approx.year)
L-model									
MLE	0.094	0.082	1.582	51	11	3.188	1.351	0.027	17859
HPD95Lo	0.030	0.026	0.914	0	0	0	0	0.006	4173
HPD95Hi	0.246	0.200	2.642	201	165	7.162	7.064	0.071	47237
M-model 1									
MLE	0.094	0.078	1.574	45	15	3.710	2.828	0.026	17922
HPD95Lo	0.034	0.026	0.914	0	0	0	0.073	0.006	3865
HPD95Hi	0.242	0.202	2.634	201	157	7.237	6.365	0.072	50251
M-model 2									
MLE	0.094	0.086	1.582	37	11	3.581	3.140	0.027	18846
HPD95Lo	0.030	0.026	0.930	0	0	0.095	0	0.007	4623
HPD95Hi	0.250	0.202	2.634	205	167	6.920	7.090	0.072	50850
M-model 3									
MLE	0.094	0.082	1.590	57	11	3.013	1.351	0.029	20269
HPD95Lo	0.034	0.030	0.898	0	0	0.163	0	0.007	4623
HPD95Hi	0.246	0.206	2.654	199	171	7.313	7.625	0.070	49428

*θ*_S_ *θ*_N_ and *θ*_A_ indicated the effective population size of the southern region, the northern region and the ancestral populations, respectively. The migration rate is indicated as backwards in time, e.g. migration parameter (*m*_N>S_) indicates migration from northern to southern population in coalescent time (i.e. from population southern to northern forward in time). *2N*_*N*_ *m*_*N*_ population migration rate from southern to northern population, *2N*_*S*_ *m*_*S*_ population migration rate from to northern to southern population. The divergence time (*T*) was scaled by geometric means of substation rates per locus (1.498 × 10^−6^)

**Table 3:** Tests of the nested demographic models

Model	Log(P)	df	2LLR	*p*
*θ*_A_ *θ*_S_ *θ*_N_ *m*_S_ = *m*_N_	7.405	1	10.38	**
*θ*_A_ *θ*_S_ *θ*_N_ *m*_S_ = 0 *m*_N_	10.03	1^a^	5.136	*
*θ*_A_ *θ*_S_ *θ*_N_ *m*_S_ *m*_N_ = 0	8.692	1^a^	7.805	**
*θ*_A_ *θ*_S_ *θ*_N_ *m*_S_ = 0 *m*_N_ = 0	−2066	2^a^	4157	**
*θ*_A_ *θ*_S_ = *θ*_N_ *m*_S_ *m*_N_	10.18	1	4.837	*
*θ*_A_ *θ*_S_ = *θ*_N_ *m*_S_ = *m*_N_	7.626	2	9.936	**
*θ*_A_ *θ*_S_ = *θ*_N_ *m*_S_ = 0 *m*_N_	6.952	2^a^	11.28	**
*θ*_A_ *θ*_S_ = *θ*_N_ *m*_S_ *m*_N_ = 0	0.9607	2^a^	23.27	**
*θ*_A_ *θ*_S_ = *θ*_N_ *m*_S_ = 0 *m*_N_ = 0	−2084	3^a^	4192	**
*θ*_A_ = *θ*_S_ *θ*_N_ *m*_S_ *m*_N_	3.166	1	18.86	**
*θ*_A_ = *θ*_S_ *θ*_N_ *m*_S_ = *m*_N_	0.5361	2	24.12	**
*θ*_A_ = *θ*_S_ *θ*_N_ *m*_S_ = 0 *m*_N_	−89.13	2^a^	203.5	**
*θ*_A_ = *θ*_S_ *θ*_N_ *m*_S_ *m*_N_ = 0	−148.7	2^a^	322.5	**
*θ*_A_ = *θ*_S_ *θ*_N_ *m*_S_ = 0 *m*_N_ = 0	−2723	3^a^	5472	**
*θ*_S_ *θ*_A_ = *θ*_N_ *m*_S_ *m*_N_	−11.45	1	48.1	**
*θ*_S_ *θ*_A_ = *θ*_N_ *m*_S_ = *m*_N_	−17.2	2	59.59	**
*θ*_S_ *θ*_A_ = *θ*_N_ *m*_S_ = 0 *m*_N_	−227.4	2^a^	480	**
*θ*_S_ *θ*_A_ = *θ*_N_ *m*_S_ *m*_N_ = 0	−65.72	2^a^	156.6	**
*θ*_S_ *θ*_A_ = *θ*_N_ *m*_S_ = 0 *m*_N_ = 0	−3210	3^a^	6445	**
*θ*_A_ = *θ*_S_ = *θ*_N_ *m*_S_ *m*_N_	−34.8	2	87.86	**
*θ*_A_ = *θ*_S_ = *θ*_N_ *m*_S_ = *m*_N_	−42.18	3	102.6	**
*θ*_A_ = *θ*_S_ = *θ*_N_ *m*_S_ = 0 *m*_N_	−236.9	3^a^	492.1	**
*θ*_A_ = *θ*_S_ = *θ*_N_ *m*_S_ *m*_N_ = 0	−171.5	3^a^	361.2	**
*θ*_A_ = *θ*_S_ = *θ*_N_ *m*_S_ = 0 *m*_N_ = 0	−3238	4^a^	6494	**

24 nested models (no migration and equal population size) were compared to the full model (significant migration and different population size; *θ*_A_, *θ*_S_, *θ*_N_, m_S_ and m_N_). ‘Log (P)’ is the posterior probability of the model given data; ‘2LLR’ = 2× (Log(P)_nested model_-Log(P)_full model_), ‘df’ is the difference in number of parameters between nested and full model except where marked with ^a^ (in which case models have distributions of 2LLR that are a mixture), ‘*p*-value’ is the probability of achieving the test statistic (2LLR) by chance under the null model. The models with *p* < 0.05 represent rejection of the models.*, *p* < 0.05; **, *p* < 0.01

*θ*_A_, *θ*_S,_ *θ*_N_ are the effective population sizes of ancestral population, southern population and northern populations, respectively. *m*_S_ and *m*_N_ are the gene flow from northern to southern lineage and southern to northern lineage, respectively

### Flowering time and temperature

**Fig. 5:**
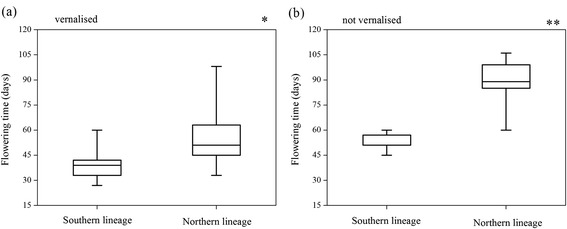
Variation in flowering time: (**a**) vernalised; (**b**) not vernalised. Each box represents the interquartile range, containing 50 % of the values and the median (horizontal line across the box). Significance levels: *p < 0.05; **p < 0.01

In the greenhouse experiment, individuals from southern regions showed significantly earlier flowering times than those from northern regions with vernalisation (*p* < 0.05) (Fig. 5a) as well as without vernalisation treatment (*p* < 0.001) (Fig. 5b). With vernalisation, the mean flowering times of the southern and northern groups were 41.06 ± 9.72 and 53.39 ± 14.99 (mean ± standard deviation) days, respectively. Without it, the average flowering times were 52.80 ± 4.73 and 90.29 ± 11.98 days, respectively. Okinawa Island samples flowered completely, whereas a substantial proportion of plants from the northern region were unable to flower in 120 days after planting (at the end of the experiment), but stayed vegetative (75 and 87.5 % in Aomori and Hokkaido Islands, respectively). Significant differences were also found within lineages for the different treatments (i.e., with and without vernalisation). This suggests that both the southern and northern populations with vernalisation exhibited earlier flowering than those without vernalisation.

**Fig. 6:**
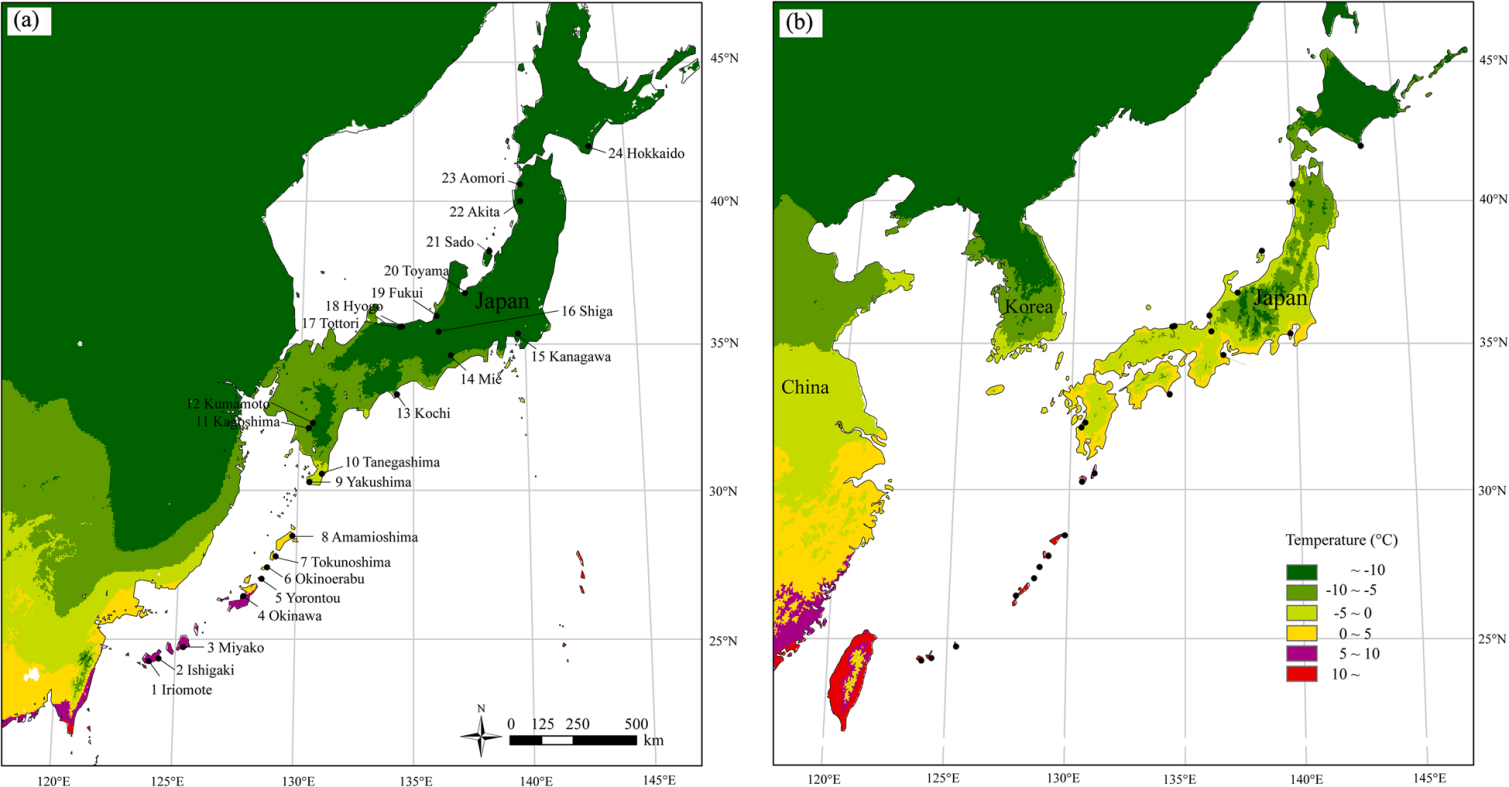
Map of the minimum temperature of the coldest month in Japan: (**a**) LGM period; (**b**) in the present

Generally, the minimum temperatures were considerably different between southern and northern regions. Southern regions have warmer temperatures than northern regions in the coldest month of the year. Northern regions (mainland Japan) experienced sub-zero temperatures 22,000 years ago, and were much colder than the southern regions (Ryukyu Islands) (Fig. 6a). In modern times, the lowest temperature in Japan occurred in Hokkaido (the northernmost island), whereas southern regions had much higher temperatures during the coldest period of time (Fig. 6b). Notably, a distinct boundary of 5 °C was found between Honshu and Ryukyu islands, both in the present and the past. Specifically, temperatures were higher than 5 °C in the southern regions but often lower than 5 °C in northern regions during the coldest month of the year.

## Discussion

### Occurrence of lineage differentiation during the LGM

The multi-locus analysis demonstrated that there was robust genetic differentiation between the southern and northern groups, consistent with previous findings [17]. However, in contrast to a previous study, the present study originally suggested that the two lineages were estimated to have diverged approximately 18,000 years ago, during the LGM period. First, divergent natural selection was proposed to be the major explanation for the lineage differentiation. Among environmental factors, temperature plays an essential role in driving the local adaptation of plants by determining the most appropriate flowering time [2]. The optimum vernalisation temperature ranges from 1–7 °C for most species [4], and 5 °C is widely used for vernalisation treatment [44–46]. Therefore, we presumed that vernalisation can occur when the local minimum temperature is lower than 5 °C but not higher than 5 °C. A latitudinal gradient of temperatures during the coldest month in Japan was found; the southern regions were basically frost-free, with temperatures above 5 °C, whereas the northern regions experienced a frost-influenced climate with minimum temperatures of 5 °C or lower as the minimum temperature, both in the LGM (Fig. 6a) and in the present (Fig. 6b). Hence, it is plausible that southern populations cannot experience cold exposure but northern populations can, which in turn may ultimately generate variation in vernalisation requirements for wild radish between southern and northern lineages. Evidence for this was provided by our greenhouse experiments, which suggested that wild radish in southern regions, exhibited a facultative vernalisation response. Cold exposure is not required for flowering, but can promote early flowering. In contrast, most northern accessions cannot flower without prior cold exposure, suggesting an obligate requirement for cold treatment, which coincides with the facultative or obligate vernalisation exhibited by *A. thaliana* [2].

Different vernalisation requirements (facultative and obligate) and different subsequent flowering times may lead to partial or complete seasonal isolation and favour prezygotic reproduction isolation between incipient lineages, and ultimately enhance and maintain genetic differentiation. Based on the variation in vernalisation requirements of wild radish, it is reasonable to presume that when the southern lineage migrates to northern regions they have earlier flowering than surrounding northern plants after cold exposure, and thus, the hybridisation of southern and northern groups would be restricted. On the other hand, when the northern lineage migrates to southern regions without prior cold exposure, they would not be able to bolt and flower. Hence, environmental heterogeneity might have resulted in different vernalisation requirements with different subsequent flowering times, which led to a reduced rate of mating between native and foreign individuals, despite the slight overlap in the flowering times of the different lineages, and in turn reinforced intra-specific differentiation. Currently, we can offer no evidence for the rate of successful migration between southern and northern regions. It would be greatly beneficial to perform reciprocal transplants across the distribution ranges and fitness trade-offs to gain a better understanding of local adaptation and reproductive isolation in wild radish.

The estimated divergence time for southern and northern lineages is consistent with geographical evidence for the existence of a directional change in the Kuroshio Current and past land configurations. During the LGM, the Kuroshio Current did not enter the Ryukyu Arc due to the appearance of a land bridge connecting Taiwan and the Ryukyu Islands [47, 48]. However, with the disappearance of the land bridge after the LGM, the main stream of Kuroshio flowed along the Ryukyu Island Arc toward the northeast, turned eastward through the Tokara Strait near Yakushima Island, and then continued north-eastward [47] (Fig. 1). Thus, it seems plausible that the directional change in the sea current influenced the seed dispersal of wild radish and lineage divergence between the southern and northern groups. In addition, effects of the Tokara Strait should not be excluded from consideration. The Tokara Strait, which is located north of the Ryukyu Islands between the Amamioshima and Tanegashima Islands (Fig. 1) [49], is a well-known phylogeographical boundary [50–52]. During the LGM, there was a 120–140 m drop in sea level, and the straits (<100 m) between the Ryukyu Islands and the main island of Japan probably disappeared. The Tokara Strait, however, persisted because its depth was more than 1,000 m. The Ryukyu Islands and mainland Japan were separated by this strait, and thus the migration of species is assumed to have been restricted [53].

### Gene flow after divergence

Migration between the southern and northern lineages of wild radish was restricted, but we found that bidirectional gene flow occurred after these populations split and that the movement of seeds from the southern to northern regions was predominant. Seeds of wild radish are reported to be water-impermeable and a large air-filled cavity allows them to float in seawater for long periods of time, during which they remain viable [54]. Thus, it seems plausible that the long-distance seed dispersal of wild radish via the Kuroshio Current is responsible for the gene flow between the southern and northern regions, serving as a migratory corridor for the transportation of these seeds. Moreover, the numerous multidirectional tributaries of sea currents surrounding the Japanese islands would contribute to the frequent transportation of wild radish seeds in several directions. Thus, the long-distance seed dispersal of this species via currents promoted gene exchange along coastlines, leading to the bidirectional gene flow between the southern and northern regions; the northward Kuroshio Current induced the higher northward gene flow.

## Conclusions

This study explicitly demonstrated divergence between the southern and northern lineages of wild radish during the LGM, despite on-going gene flow sustained by the high dispersability and longevity of seeds. Environmental factors such as temperature and geographical barriers were responsible for the genetic differentiation. Divergent natural selection can lead to different vernalisation requirements (facultative and obligate) and different subsequent flowering times, which may suppress the reproductive success of immigrants and ultimately drive and reinforce genetic differentiation. In addition, the lineage differentiation may also be related to the geographic barrier formed by the effects of sea currents and past land configurations. Consequently, this study is the first to provide a comprehensive and clear description of the demographic history of the East Asian wild radish, incorporating genetic diversity, life strategies and habitat temperature variables. More importantly, the different vernalisation requirements of wild radish will provide valuable information for developing new cultivars that are better adapted to their respective habitat climates. The present study provides new insights into the maintenance of divergence in spite of gene flow in coastal plants in different habitats.

### Availability of supporting data

The original haplotype sequences of 14 nuclear loci were deposited in GenBank (Accession numbers: KU949773–KU949946).

### Ethical approval

Not required.

### Consent to publish

Not required.

## Additional files

Additional file 1: Table S1. Locus information and list of the primers (*: internal primers). (DOCX 20 kb) Additional file 2: Figure S1. The distribution of model parameters (∆*K*) and log-likelihood of the data (Ln*P*(D)) based on structure analysis. (PDF 51 kb)

## Abbreviations

ANOVA: analysis of variance
bp: base pairs
cpDNA: chloroplast DNA
CTAB: cetyltrimethylammonium bromide
ESS: effective sample size
HKA: Hudson-Kreitman-Aguade
HPD: highest probability density.
IM: isolation-with-migration
IS: infinite-sites
LGM: Last Glacial Maximum
MCMC: Markov chain Monte Carlo
MLEs: maximum likelihood estimates
PCoA: principal coordinate analysis
PCR: polymerase chain reaction
*χ*2: Chi-squared
