# Supplementary material for: Lineage isolation in the face of active gene flow in the coastal plant wild radish is reinforced by differentiated vernalisation responses
Source: BMC Evol Biol. 2016 Apr 16;16:84. doi: 10.1186/s12862-016-0655-7 (PMC4833902; doi:10.1186/s12862-016-0655-7)
Supplement: Additional file 1: Table S1. — Locus information and list of the primers (*: internal primers). (DOCX 20 kb) [file 12862_2016_655_MOESM1_ESM.docx]

**Table S1** Locus information and list of the primers (*: internal primers)

| Locus | TAIR10 Top Hit | Arabidopsis TAIR10 description | Primer name | Sequences (5′-3′) |
| --- | --- | --- | --- | --- |
| *PHYA** | AT1G09570.1 | Phytochrome A | PHYA-F | GGCCATCATCCAAAACCCAA |
|  |  |  | PHYA-R | CCCCTCCATCATCTTCCTTGT |
| *PHYB* | AT2G18790.1 | Phytochrome B | PHYB-F | CAACATTGTCGGGGTGTGTT |
|  |  |  | PHYB-R | AGCCTCAAGACACGGAATCT |
| *PHYC* | AT5G35840.1 | Phytochrome C | PHYC-F | ACAGGCCAGAAGACGCTT |
|  |  |  | PHYC-R | ACTTGTTGGCGGAGATATGC |
| *PHYE** | AT4G18130.1 | Phytochrome E | PHYE-F | TGGTGTTTGCTTCGTTGGT |
|  |  |  | PHYE-R | TCCGATGACTTTCCCTTCAGT |
| *TBL19** | AT5G15900.1 | Trichomes birefringence-like 19 | TBL19-F | ATGGAGCTTCTACACTCTGC |
|  |  |  | TBL19-R | GACTGCATGTGGTTTCTGCT |
| *TBL21* | AT5G15890.1 | Trichomes birefringence-like 21 | TBL21-F | CCATGTGGTCACCGTTTCTAG |
|  |  |  | TBL21-R | CATTTCTTAAACTCACCTCTGCG |
| *MYB29** | AT5G07690.1 | MYB domain protein 29 | MYB29-F | TGCCTCTAACCCTGAACCAG |
|  |  |  | MYB29-R | TCCTCGGCTGCATCGTTAT |
| *COL4* | AT5G24930.1 | CONSTANS-like 4 | COL4-F | CGGCGTTTCTATGTCTCAGC |
|  |  |  | COL4-R | GCAGTTGTGTCCGTACGTAG |
| *COL5** | AT5G57660.1 | CONSTANS-like 5 | COL5-F | CTCTGCGAAGTCTGCGAAC |
|  |  |  | COL5-R | CTGTCTGCTCCGAAAGTGT |
| *CHI* | AT2G43570.1 | Chitinase, putative | CHI-F | TCCTGTGCCACATTGAGGA |
|  |  |  | CHI-R | CTCCAGGTTCAACTCCAAGC |
| *VEL2** | AT2G18880.1 | VEL2 \| vernalization5/VIN3-like | VEL2-F | TCGAGGATGTTGATGCGACT |
|  |  |  | VEL2-R | AGAATGTACGAATGAGCTGGTC |
| *LFY* | AT5G61850.1 | Floral meristem identity control protein LEAFY (LFY) | LFY-F | TCACTGTTACGCACTCCACT |
|  |  |  | LFY-R | ATCCCTCGCAGCTAATACCG |
| *CRY2** | AT1G04400.1 | Cryptochrome 2 | CRY2-F | GCTAGAGAACGAGGCGGA |
|  |  |  | CRY2-R | CCAACGGATACCCAGTCCT |
| *CRY3* | AT5G24850.1 | Cryptochrome 3 | CRY3-F | CGTGTCTCTCGTCTCCTCTC |
|  |  |  | CRY3-R | CGGTTTTAGGGAAGGCGAAG |
