# Supplementary figures and images for: Lineage isolation in the face of active gene flow in the coastal plant wild radish is reinforced by differentiated vernalisation responses
Source: BMC Evol Biol. 2016 Apr 16;16:84. doi: 10.1186/s12862-016-0655-7 (PMC4833902; doi:10.1186/s12862-016-0655-7)

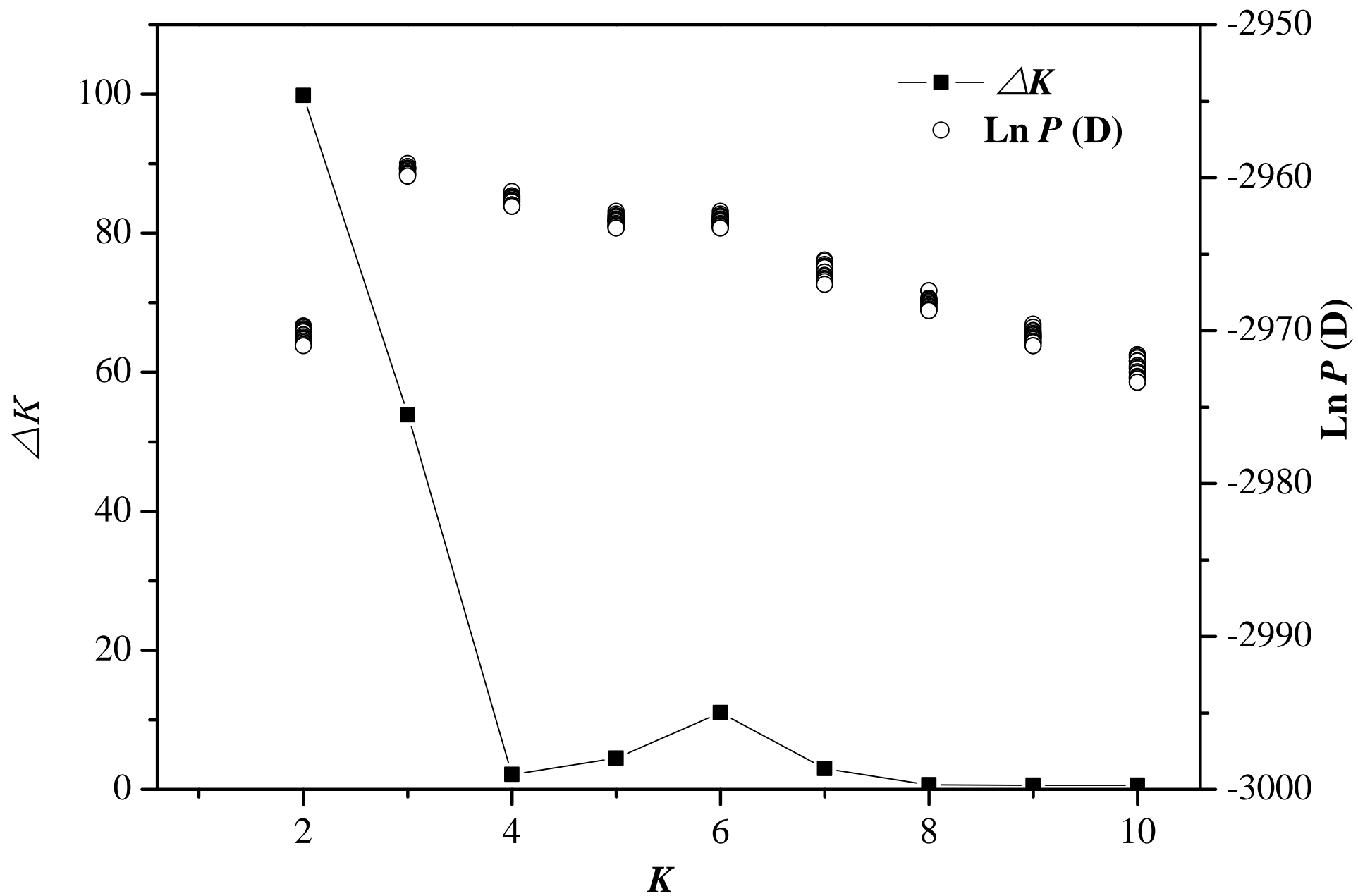

Supplement: Additional file 2: Figure S1. — The distribution of model parameters (∆K) and log-likelihood of the data (LnP(D)) based on structure analysis. (PDF 51 kb) [file 12862_2016_655_MOESM2_ESM.pdf]
